# El enfoque de Orientación Cognitiva al Desempeño Ocupacional Diario en discapacidades con origen en la infancia

**DOI:** 10.1111/dmcn.16401

**Published:** 2025-07-04

**Authors:** Hortensia Gimeno, Helene Polatajko

**Affiliations:** ^1^ Barts Bone and Joint Health, Blizard Institute Queen Mary University of London London UK; ^2^ The Royal London Hospital and Tower Hamlets Community Children's Therapy Services Barts Health NHS Trust London UK; ^3^ Department of Occupational Science and Occupational Therapy, Rehabilitation Sciences Institute, Faculty of Medicine University of Toronto Toronto Canada

## Abstract

El enfoque de Orientación Cognitiva al Desempeño Ocupacional Diario (Cognitive Orientation to daily Occupational Performance en inglés o CO‐OP), es una intervención orientada a objetivos centrada en la participación. Diseñada para mejorar el desempeño abordando objetivos personales importantes para los niños y sus familias. Con sus orígenes en 2001, el enfoque CO‐OP implica objetivos funcionales elegidos por el cliente, identificando problemas de desempeño a través de un proceso conocido como análisis del desempeño dinámico (ADD) y guiando el descubrimiento de estrategias cognitivas para mejorar la adquisición de habilidades. Todo este proceso se enmarca dentro de un marco de resolución de problemas. Los objetivos del enfoque son la adquisición de habilidades, el uso de estrategias, la generalización y la transferencia del aprendizaje. Desarrollado dentro de un marco de investigación, una revisión de la literatura indica que la investigación sobre CO‐OP se ha ampliado, documentando su uso en diversas poblaciones pediátricas, incluidos niños con trastornos del neurodesarrollo, parálisis cerebral y trastornos del movimiento y abordando una variedad de objetivos funcionales centrados en el cliente. En esta revisión ilustramos el desarrollo repetido del enfoque CO‐OP desde diseños experimentales de caso único hasta ensayos clínicos aleatorizados para evaluarlo. Las medidas para evaluar resultados más comunes son la Medida Canadiense de Desempeño Ocupacional (COPM) y la Escala de Calidad del Desempeño (PQRS). Se discuten los avances metodológicos, las limitaciones y una exploración inicial de los mecanismos de acción, proporcionando una base para futuras investigaciones y aplicaciones clínicas. Las recomendaciones incluyen el uso de medidas consistentes, estudios longitudinales robustos, investigación de implementación y análisis económicos dentro de la salud.

**Abbreviations**
ADD: Análisis dinámico del desempeño
CO‐OP: Cognitive Orientation to daily Occupational Performance
PQRS: Escala Calidad del Desempeño
RCT: Ensayo clínico aleatorizado
TDC: Trastorno del desarrolo de la coordinacion

El enfoque de Orientación Cognitiva para el Desempeño Ocupacional Diario (Cognitive Orientation to daily Occupational Performance en inglés o CO‐OP), ^1^ fue introducido en 2001 como una intervención innovadora para niños con trastorno del desarrollo de la coordinación (TDC). En el momento de su desarrollo, el enfoque CO‐OP contrastaba con los modelos tradicionales al centrarse directamente en la adquisición de habilidades mediante el uso de estrategias cognitivas, sin abordar específicamente las deficiencias subyacentes. A lo largo de más de dos décadas, CO‐OP se ha expandido significativamente, demostrando ser relevante para diversas poblaciones, incluyendo niños con parálisis cerebral (PC), autismo y lesiones cerebrales adquiridas.

En esta revisión narrativa examinamos el desarrollo del enfoque CO‐OP a través de múltiples ciclos de investigación, desde diseños de caso único y estudios piloto, hasta ensayos controlados aleatorios (RCTs) y revisiones sistemáticas. Exploramos el uso del enfoque población con diferentes diagnósticos, utilizando lo que se conoce como estrategias específicas de dominio para abordar objetivos variados, destacando su adaptabilidad en población infantil con diferentes discapacidades. Se ofrecen recomendaciones para investigaciones futuras.

## EL ENFOQUE CO‐OP

El enfoque CO‐OP, presentado por primera vez en la literatura 2001^2^ como una intervención novedosa para niños con TDC, es, ante todo, un enfoque orientado a objetivos diseñado para mejorar el desempeño. En contraste con muchos enfoques de rehabilitación que buscan abordar deficiencias subyacentes y componentes del desempeño, como reducir el tono muscular o mejorar el equilibrio o las habilidades motoras finas, los enfoques orientados a objetivos se centran explícitamente en lograr objetivos funcionales específicos.^3^ CO‐OP comienza con la identificación de objetivos, que luego se convierten en el foco de la intervención hasta que estos se logran.^4^, ^5^, ^6^, ^7^

Otros términos, como entrenamiento orientado a tareas o específico a tareas, también se utilizan para describir intervenciones que se enfocan en problemas específicos de desempeño en actividades, en lugar de abordar deficiencias subyacentes y limitaciones funcionales.

CO‐OP se diferencia de otras intervenciones orientadas a objetivos o específicas a tareas en que involucra al cliente/niño como un colaborador activo en el proceso terapéutico de resolución de problemas para alcanzar sus objetivos personales, comenzando con los objetivos personales que los niños, jóvenes y sus familias identifican como importantes en su vida cotidiana. CO‐OP es único porque utiliza un enfoque no directivo para la adquisición de habilidades llamado descubrimiento guiado, que permite la resolución de problemas, elemento clave de la intervención, y tiene como objetivo específico la generalización y transferencia de habilidades.

CO‐OP es una intervención individualizada y centrada en el cliente, fundamentada en teorías de aprendizaje cognitivo y motor. El enfoque CO‐OP derivó del trabajo de Meichenbaum y de los principios de la terapia cognitivo‐conductual, donde se enseña a los clientes una estrategia de resolución de problemas y auto‐instrucción verbal para abordar patrones de pensamiento poco útiles.^8^ En CO‐OP, la estrategia de resolución de problemas se ha adaptado para abordar problemas en las áreas de actividad y participación. Con su base teórica en enfoques de aprendizaje y cognición, el aprendizaje de nuevas habilidades se considera un ejercicio de resolución de problemas que debe ocurrir en un contexto apropiado (es decir, en el hogar o en la escuela) para facilitar la adquisición adecuada de habilidades, su generalización y transferencia. CO‐OP se fundamenta en una variedad de perspectivas teóricas, las cuales contribuyen al enfoque terapéutico.

El enfoque utiliza un marco compuesto por siete características claves, cinco de las cuales son fundamentales para desarrollar, aplicar y generalizar estrategias cognitivas que permitan resolver problemas de desempeño cotidiano.

Los cinco elementos fundamentales del enfoque CO‐OP son los siguientes. (1) Objetivos funcionales elegidos por el cliente: Estos objetivos son establecidos por el niño/joven, por lo que son significativos y relevantes para ellos. (2) Análisis del desempeño dinámico (ADD): Consiste en observar y analizar detenidamente cómo los niños y jóvenes realizan tareas, identificando interrupciones en el desempeño y estrategias potenciales que puedan ser utilizadas. Finalmente, los niños y jóvenes aprenden a llevar a cabo su propio análisis de su desempeño dinámico. Este proceso dinámico repetido se basa en un análisis de la interacción entre la persona, la tarea y el entorno. (3) Descubrimiento guiado: Es el proceso mediante el cual se resuelven los problemas de desempeño identificados en el de ADD. El descubrimiento guiado respalda la identificación de problemas y estrategias de desempeño, y a su vez, la adquisición de habilidades. En lugar de proporcionar instrucciones directas, el terapeuta del enfoque CO‐OP facilita que los clientes encuentren sus propias soluciones a sus propios problemas, utilizando preguntas, entrenamiento trabajando solo una cosa a la vez y estando en sintonía con el cliente. (4) Uso de estrategias cognitivas: Se emplean dos tipos de estrategias cognitivas: una estrategia global y estrategias específicas conocidas como estrategias de dominio. Estas apoyan la adquisición de habilidades, la generalización y la transferencia. La estrategia global fomenta la metacognición enseñando el mnemotécnico: ‘Objetivo‐Plan‐Acción‐Comprobación’ (Goal‐Plan‐Do‐Check en inglés), diseñado para apoyar la resolución de problemas. Este se utiliza de manera iterativa a lo largo de la intervención. Objetivo ‐ Define lo que el cliente quiere lograr; Plan ‐ Desarrolla un plan para alcanzar el objetivo; Hacer ‐ Ejecuta el plan; Verificar ‐ Evalúa la ejecución del plan y su efectividad, apoyando ajustes o nuevos planes, según sea necesario. Por otro lado, las estrategias de dominio específico como su nombre indica, son específicas para los problemas producidos durante el desempeño y están diseñadas para superarlos. Estas se utilizan sólo durante un tiempo breve y son específicas/relevantes para la tarea, el niño y el entorno. (5) Principios facilitadores: Son los fundamentos teóricos en los que el terapeuta se apoya para fomentar la adquisición de habilidades, la generalización y la transferencia. En combinación con el ADD y el descubrimiento guiado, los cuatro principios facilitadores apoyan a los niños y jóvenes en la resolución de problemas y el logro de los objetivos que se proponen. Uno de los principios más importantes es promover la generalización y la transferencia.

El enfoque incluye dos características adicionales que son algo flexibles en su implementación y ejecución:

(6) Participación de personas significativas: Al trabajar con niños y jóvenes, la participación de los padres y otras personas significativas es esencial para que se conviertan en colaboradores activos de la intervención. Al aprender cómo implementar el ADD y el descubrimiento guiado, los padres y personas significativas están capacitados para apoyar el uso de las características clave de la intervención en la vida cotidiana. Esto es crucial para la generalización y transferencia del aprendizaje más allá de las sesiones de terapia. Las personas significativas actúan como un puente entre la intervención terapéutica y la realidad diaria del niño las 24 horas del día, los 7 días de la semana. (7) Formato intervención: El formato de la intervención se divide en fases principales. La fase 1 es la de preparación, la cual incluye el establecimiento de objetivos. La fase 2 es la de adquisición, la cual consta generalmente de 10 sesiones de intervención. En la primera sesión se enseña la estrategia global ‘Objetivo‐Plan‐Acción‐Comprobación’ (Goal–Plan–Do–Check en inglés), y en las sesiones 2 a 10 se fomenta que el niño utilice esta estrategia global, aprenda a realizar el ADD y descubra el uso de estrategias de dominio específico. La fase 3 es de cierre y reevaluación y corresponde a la última sesión, donde se revisan los objetivos y se evalúan los progresos logrados.

Los cuatro objetivos clave de la intervención CO‐OP son (1) adquisición de habilidades, (2) uso de estrategias cognitivas, (3) generalización y (4) transferencia del aprendizaje. CO‐OP involucra activamente al cliente en generar, probar y refinar sus propias estrategias para alcanzar sus propios objetivos. Este aspecto de CO‐OP está diseñado para apoyar la transferencia y la generalización a otros contextos y otros objetivos, con beneficios que se extienden más allá de la duración de la intervención terapéutica. El papel del terapeuta de CO‐OP es ayudar a los clientes a emplear una estrategia de resolución de problemas que les permita desarrollar sus propias estrategias efectivas.

## CONOCIMIENTOS ACTUALES SOBRE EL USO DEL ENFOQUE

A diferencia de muchas intervenciones de rehabilitación, el enfoque CO‐OP se originó en el campo de la investigación y comenzó con estudios de prueba de concepto utilizando diseños de caso único ^9^ y series de casos^10^ para tratar a niños con TDC. El enfoque, originalmente denominado “guía auto‐verbal”, fue renombrado como CO‐OP cuando los análisis de las sesiones de intervención revelaron que las mejoras observadas en esa población eran por el uso de una variedad de estrategias cognitivas y no sólo la guía auto‐verbal, en el contexto de uso de estrategias de resolución de problemas.^11^ Las investigaciones iniciales fueron seguidas por estudios de réplica y seguimiento. Finalmente, se realizó un ensayo piloto controlado aleatorio (RCT).^2^ La evidencia que respalda el uso del enfoque en niños con TCD impulsó la investigación del enfoque en otras poblaciones.^12^ Una búsqueda rápida completada en marzo de 2024 utilizando los términos ‘CO‐OP’, ‘Cognitive Orientation to Occupational Performance’ y ‘Cognitive Orientation to daily Occupational Performance’ resultó en 136 artículos tras eliminar duplicados. Una vez seleccionados los artículos, 38 incluían a niños y jóvenes, y contenían datos primarios reportados. Esto muestra que CO‐OP se ha expandido enormemente desde su introducción en 2001, no sólo en el número de estudios disponibles, sino también en varios aspectos importantes. Muchos de los estudios encontrados en la literatura son estudios de intervención que evalúan su uso con diferentes poblaciones, utilizando una variedad de diseños de investigación y enfoques para medir los resultados, diferentes formatos de presentación (presentación individual frente a grupal) y dosis, y abordando una amplia gama de objetivos, con una variedad de tipos de estrategias diferentes y enfoques para evaluar la generalización y la transferencia.

CO‐OP se ha utilizado en varias poblaciones de diferentes edades, desde niños de 5 años de edad^13^, ^14^ hasta adultos mayores que han experimentado accidentes cerebrovasculares o deterioro cognitivo leve.^15^ Existe una revisión sistemática de estudios sobre el uso de CO‐OP en accidentes cerebrovasculares.^15^ Aquí nos centramos en la literatura sobre CO‐OP tal como se aplica a niños y jóvenes.

El uso de CO‐OP con niños y jóvenes con trastornos del neurodesarrollo ha sido recientemente sintetizado en una revisión sistemática.^16^ El enfoque también se ha usado con niños con otras afecciones neurológicas, como parálisis cerebral (PC)^17^, ^18^, ^19^, ^20^, ^21^ y trastornos más severos del movimiento^22^, ^23^, ^24^ .Es la aplicación de CO‐OP en personas con trastornos neurológicos más evidentes, primero en pacientes con accidentes cerebrovasculares ^25^, ^26^, ^27^, ^28^, ^29^ y después en niños y jóvenes con trastornos del movimiento hipercinéticos, incluyendo distonía^22^, ^23^, ^24^, ^30^ lo que hizo cuestionar nuestras premisas y paradigmas iniciales en torno a la adquisición de habilidades motoras.

La Tabla 1 muestra los diferentes grupos de población pediátrica investigados para evaluar la aplicación del enfoque y los diseños utilizados.

**TABLA 1:** Resumen de estudios CO‐OP en niños y jóvenes (datos primarios)

Condición	Estudios	Rangos de edad	Tipos de diseño	Tamaño de muestra	Dosificación	Formato
Lesión cerebral adquirida (incluyendo función ejecutiva)	*n* = 3	6–16 años	Diseño cuasi‐experimental pre‐post (*n* = 1) SCED con réplicas (*n* = 2)	10 2 y 12	10–14 sesiones	Individual
TDAH	*n* = 1	7–12 años	SCED con réplicas	6	12 sesiones	Individual
Autismo	*n* = 4	5–12 años	Estudios de caso (*n* = 4)	7	10 sesiones	Individual
TDC incluyendo TDC&TDAH	*n* = 17	5–16 años	SCED (*n* = 2) Piloto RCT (*n* = 2) RCTs (*n* = 2) Pre‐post‐comparación de grupos (*n* = 6) Estudios de caso (*n* = 5)	8 8 y 20 22 y 41 20, 12, 18, 8, 4 1–4	10 sesiones (*n* = 10) 8–9 sesiones (*n* = 2) 7 sesiones de 2 horas (*n* = 1) 12 sesiones (*n* = 1) Intensidad 2x50 minutos al dia por 4 días (*n* = 1) 9.5 horas en 5 días más trabajo grupal (*n* = 1) 20 1 hour (*n* = 1)	Individual (*n* = 13) Grupo (*n* = 4)
Syndrome de Down	*n* = 2	7–19 años	SCED (*n* = 1) RCT (*n* = 1)	6 12	7–8 sesiones 10 sesiones	Individual
Parálisis Cerebral, Espina Bifida, y trastornos de movimiento	*n* = 11	4–28 años	SCED (*n* = 3) Piloto RCT (*n* = 1) RCT (*n* = 3) Estudios de caso único (*n* = 1) Diseño cruzado (*n* = 1)	31 18 45, 32, 38 10 12	10 sesiones a la semana o dos veces por semana 45–60 minutos	Individual (*n* = 10) Grupo (*n* = 1)
Otros—retraso intelectual	*n* = 1		Diseño cuasi‐experimental pre‐post	30	36 sesiones	Individual

Abreviaciones: TDAH, Trastorno por déficit de atención e hiperactividad; CO‐OP, TDC, trastorno del desarrollo de la coordinación; RCT, randomized controlled trial; SCED, single‐case experimental design.

Típicamente, se han realizado estudios pequeños cada vez que se prueba el enfoque CO‐OP en una nueva población para proporcionar una prueba de concepto. Existe una corriente particular en el uso de estudios de caso único con múltiples líneas base y replicaciones en muchos de estos estudios.^23^, ^24^, ^31^, ^32^ Se reconoce que el uso de estudios de caso único, particularmente en trastornos raros y otras poblaciones, es preferible a realizar ensayos controlados aleatorios (RCTs) pequeños y sin suficiente poder estadístico.^33^ Los estudios de caso único permiten explorar los resultados individuales, como en el caso de varios participantes que logran sus objetivos, lo que amplía nuestra comprensión sobre las mejores respuestas posibles. .

La mayoría de los estudios realizados hasta la fecha han explorado la eficacia (o eficacia preliminar) de CO‐OP en diferentes poblaciones cuando se aplica en un entorno de investigación controlado. La efectividad de la intervención con CO‐OP entregada por clínicos (en lugar de en un entorno de investigación) ha sido explorada en un pequeño número de estudios ^17^, ^23^ y, en adultos, en un estudio de ciencia de implementación llevado a cabo en cinco unidades de rehabilitación para pacientes hospitalizados con accidente cerebrovascular.^34^

### Diseño y medidas

La Tabla 1 muestra los estudios con datos primarios de CO‐OP con su diseño de estudio y tamaño de muestra. La mayoría de los estudios han implementado CO‐OP a nivel individual, mientras que cinco estudios lo han implementado en formato grupal.^35^, ^36^, ^37^, ^38^, ^39^ Al revisar los 38 estudios con datos primarios sobre CO‐OP, los objetivos se midieron de manera subjetiva en la mayoría de los estudios (n = 35, 92%) utilizando la Medida Canadiense del Desempeño Ocupacional (Canadian Occupational Performance Measure, COPM, en inglés)^40^ y en un menor número de estudios (n = 23, 61%) con la Escala de la Calidad del Desempeño (Performance Quality Rating Scale, PQRS, en inglés) en sus diferentes formas.^41^, ^42^ La aplicación de PQRS en personas con distonía, junto con las consultas a niños, jóvenes y familias para comprender y analizar los datos recopilados (parte de clientes y trabajo de investigación publicado y facilitado por Gimeno, H) , reveló la necesidad de garantizar que la herramienta fuera individualizada y que no penalizaran los movimientos involuntarios.^42^ Por lo tanto, en estos casos usamos el término PQRS‐individualizada.

### Formatos de ejecución y dosis

Para los estudios de intervención de CO‐OP entregados de forma individual, la mayoría de los estudios (n = 21, 55%) se llevaron a cabo en 10 sesiones de una hora semanales.^2^, ^13^, ^14^, ^17^, ^23^, ^24^, ^43^, ^44^, ^45^, ^46^, ^47^, ^48^, ^49^, ^50^, ^51^, ^52^, ^53^, ^54^, ^55^, ^56^, ^57^ Tres estudios aplicaron entre 7 y 9 horas,^58^, ^59^, ^60^ cuatro estudios entregaron 12 sesiones,^20^, ^31^, ^61^, ^62^ y hubo dos estudios con 14 sesiones.^32^, ^63^ La dosis entregada en formatos grupales fue variable, con dos estudios informando que proporcionaron ocho sesiones,^37^, ^39^ 10 sesiones en otros dos grupos,^36^, ^38^ y un estudio entregando 20 sesiones de una hora.^35^

### Amplia variedad de objetivos

Diferentes estudios han abordado una gran variedad de objetivos elegidos por los clientes. Los objetivos individuales se reportan en 23 estudios, y seis estudios categorizaron/tematizaron los objetivos en áreas. Objetivos específicos (n = 180) se reportaron en 29 de los estudios primarios identificados, distribuidos en las siguientes poblaciones: autismo (n = 4, siete niños y jóvenes), síndrome de Down (n = 2, 11 niños y jóvenes), TDC (incluyendo trastorno por déficit de atención/hiperactividad) (n = 12, 74 niños y jóvenes), lesión cerebral adquirida y función ejecutiva (n = 3, 11 niños y jóvenes), parálisis cerebral (CP) y espina bífida (n = 6, 58 niños y jóvenes), y trastorno del movimiento hipercinético (n = 2, 19 niños y jóvenes). En general, los objetivos están categorizados en los estudios que informan sobre los objetivos trabajados por los niños y jóvenes durante las sesiones de CO‐OP. Un resumen del tipo de objetivos por grupo poblacional está representado en la Tabla 2. Los objetivos descritos en estos estudios pueden ser categorizados en: (1) auto‐cuidado (35% de objetivos); (2) habilidades de la vida diaria (12% de los objetivos); (3) objetivos relacionados con la escuela (23% de objetivos), siendo la escritura uno de los objetivos más frecuentes, identificadas 59 veces; (4) actividades recreativas y deportivas (27% de los objetivos); (5) planificación y organización (8% de los objetivos); y (6) actividades sociales (2% de los objetivos). Se observaron algunas diferencias en el tipo de objetivos según el grupo poblacional. Mientras que las actividades recreativas y deportivas fueron las más frecuentemente reportadas para niños y jóvenes con TDC (41% de los objetivos), los objetivos relacionados con la escuela fueron más frecuentes en estudios que incluyeron a niños autistas (34%) y con lesión cerebral adquirida y función ejecutiva (45%). El autocuidado fue el tema principal de objetivos identificado por niños y jóvenes con trastorno del movimiento hipercinético (53%), parálisis cerebral y espina bífida (38%), síndrome de Down (50%) y autismo (34%).

**TABLA 2:** Tipo y número de objetivos identificados en estudios.

Condición y número de estudios (número de sujetos)	Tipos de objetivos (n)/número de objetivos						
	Auto‐cuidado	Actividades de la vida diaria	Objetivos relacionados con el colegio	Actividades de ocio y deportes	Planificación y organización	Actividades sociales	Otros
Autismo 4 (7)	10/29 (34%)	2/29 (7%)	10/29 (34%)	1/29 (3%)	3/29 (10%)	3/29 (10%)	0
Síndrome de Down 2 (11)	8/16 (50%)	2/16 (13%)	0	6/16 (37%)	0	0	0
TDC (includo TDAH) 12 (74)	51/226 (23%)	6/226 (3%)	59/226 (26%)	92/226 (41%)	11/226 (5%)	0/226	4/226 (2%)
Lesión cerebral adquirida and FE 3 (11)	2/31 (6%)	2/31 (6%)	14/31 (45%)	7/31 (23%)	5/31 (16%)	1/31 (3%)	0
PC and espina bífida 6 (58)	84/221 (38%)	33/221 (15%)	36/221 (16%)	31/221 (14%)	23/221 (10%)	8/221 (4%)	6/221 (3%)
Trastornos de movimiento hipercinético 2 (19)	30/57 (53%)	20/57 (35%)	2/57 (4%)	5/57 (9%)	0/57	0/57	0/57
Total 29 (180)	185/523 35%	65/523 12%	121/523 23%	142/523 27%	42/523 8%	12/523 2%	10/523 2%

Abreviaturas: TDAH, Trastorno por Déficit de Atención e Hiperactividad; PC, parálisis cerebral; TDC, trastorno del desarrollo de la coordinación, FE, Funciones ejecutivas.

## USO DE ESTRATEGIAS

A través del análisis de las sesiones de intervención, se han identificado las clases de estrategias específicas del dominio utilizadas por diferentes poblaciones en estudios que emplean el enfoque con niños con TDC, síndrome de Asperger, trastornos del movimiento y personas con accidente cerebrovascular.^11^, ^31^, ^44^, ^52^, ^54^, ^64^, ^65^ Estas estrategias específicas del dominio (Elemento clave 4, descrito anteriormente) no se enseñan a los participantes sino que los participantes descubren estas estrategias cuando los terapeutas emplean el descubrimiento guiado.

El primer grupo de estrategias observadas fue categorizado a partir de sesiones de intervención grabadas con niños con TDC, lo que resultó en siete categorías,^11^ resumidas en la Tabla 2 (estrategias números 1–7). Desde entonces, a partir del trabajo con otras poblaciones, han surgido nuevas clases de estrategias específicas del dominio, como la relajación y la imaginación, observadas en estudios de CO‐OP con clientes con trastornos del movimiento y/o PC^31^ y accidentes cerebrovasculares.^64^ Estas estrategias están incluidas en la Tabla 3 (estrategias números 8–9). Nuevas estrategias específicas del dominio fueron analizadas a partir de 40 horas de grabaciones en video de intervenciones de CO‐OP realizadas con niños y jóvenes con trastornos del movimiento hipercinéticos, incluyendo distonía, identificándose estrategias novedosas.^31^ Estas estrategias novedosas observadas durante las sesiones de tratamiento de CO‐OP incluyeron distracción, regulación emocional, auto‐guía mental, y atención enfocada externa e interna, como se describe en la Tabla 3 (estrategias números 10–14).

**TABLA 3:** Estrategias cognitivas conocidas de CO‐OP.

Estrategia	Descripción
**1. Posición corporal**	Verbalización de atención a, o cambio de, la posición del cuerpo, completo o parcial, en relación con la tarea.
**2. Atención al hacer**	Verbalización para indicar la atención al acto de realizar la tarea.
**3. Especificación/modificación de la tarea**	Discusiones sobre los detalles o modificaciones de la tarea o partes de la tarea que facilitan el desempeño.
**4. Complementar el conocimiento de la tarea**	Verbalización de detalles de una tarea, cosas que no pueden descubrirse o que no son centrales para el enfoque de atención.
**5. Sensación del movimiento**	Verbalización de atención a la sensación de un movimiento particular mientras se lleva a cabo.
**6. Mnemotecnia verbo‐motora**	El cliente asigna un nombre a la tarea o a un componente de la tarea o posición corporal que evoca una imagen mental para guiar el desempeño motor.
**7. Guion verbal automático**	Un patrón fijo de palabras utilizado para guiar una secuencia.
**8. Imaginación**	Un proceso activo durante el cual se reproduce mentalmente una acción específica sin realizar movimientos reales.
**9. Relajación**	Un intento de devolver el sistema de uno mismo a un estado de tranquilidad o disminuir el esfuerzo.
**10. Distracción (pasiva, activa, aumentando la carga cognitiva)**	Algo que desvía la atención del cliente del enfoque en la tarea o los movimientos corporales asociados con la tarea.
**11. Regulación emocional**	Un recordatorio para evocar comportamientos o visualizaciones que reduzcan la ansiedad o tensión, ya sea antes o durante la realización de la tarea.
**12. Guía del sentido de uno mismo**	Cualquier verbalización para la formación de imágenes mentales o semejanzas de personas/lugares/cosas que apoyen el desempeño de la tarea.
**13. Atención enfocada externamente**	Cualquier verbalización para dirigir y mantener la atención hacia un objeto o ubicación relacionada con la tarea, fuera del cuerpo (por ejemplo, el destino final o el objeto que se manipula y transporta).
**14. Atención enfocada internamente**	Cualquier verbalización para dirigir o mantener la atención al cuerpo, completo o en parte, que está siendo utilizado activamente durante la realización de la tarea.

### Enfoque para evaluar generalización y transferencia

La generalización más allá de las tareas entrenadas y la transferencia se ha informado en una revisión de alcance reciente^66^ que contiene estudios incluidos en la revisión de alcance anterior (con una pregunta de investigación diferente).^67^ La evidencia de transferencia se midió utilizando cuatro indicadores diferentes que incluyen (1) Transferencia a objetivos no trabajados durante CO‐OP; (2) puntuaciones de otras evaluaciones estandarizadas de actividades, habilidades o componentes del desempeño ; (3) puntuaciones de resultados validados reportados por pacientes; y (4) informes anecdóticos. La mayoría de los estudios abordaron la transferencia (n = 25), con 16 evaluando la transferencia explícitamente y nueve abordando la transferencia con uno de los indicadores mencionados anteriormente. Los autores de la revisión destacan la variabilidad en los enfoques para medir la transferencia y las medidas y definiciones utilizadas, lo que limita la comparación entre estudios.

## CRITICA DE LA EVIDENCIA DISPONIBLE

Una limitación importante en muchos de los estudios disponibles es el pequeño tamaño de muestra, con sólo un poco más de un tercio de los estudios incluyendo más de 10 pacientes (mediana = 8.5, rango 1–45). Típicamente, se realizan estudios pequeños cada vez que se prueba el enfoque CO‐OP en una nueva población, en un esfuerzo por proporcionar una prueba de concepto. El principal grupo de niños en los que CO‐OP ha sido validado y se ha establecido como prueba de concepto (y más allá para algunos grupos) incluye TDC, autismo, parálisis cerebral (PC), lesión cerebral adquirida, función ejecutiva y trastornos hipercinéticos de inicio en la infancia, incluyendo trastornos raros de distonía genética.

La literatura muestra que el enfoque de investigación es predominantemente el uso de estudios de caso único con replicas. Aunque se ha informado del uso de estudios de caso único con múltiples líneas base en 10 estudios de CO‐OP, no todos proporcionaron suficientes replicas (mínimo de N‐de‐1 con tres replicas)^13^, ^20^, ^23^, ^24^, ^31^, ^32^, ^45^, ^59^ según guías establecidas.^68^ Esto ha permitido el desarrollo de este enfoque con diversas poblaciones, incluyendo trastornos raros.

Sin embargo, se debe prestar más atención a enfoques más robustos para establecer la eficacia mediante un ensayo definitivo (Recomendación 1).

Un problema significativo en la mayoría de los estudios ha sido la posibilidad de sesgo del evaluador. Los estudios usaron o un solo evaluador o no demostraron (o informaron) la fiabilidad inter‐evaluador. Aunque es posible hacer una evaluación ciega con PQRS, esto no siempre se ha descrito en las publicaciones. Dos estudios especificaron claramente que no se logró la valoración ciega de PQRS.^69^, ^70^ En los estudios restantes, no se proporciona información que permita determinar si se utilizó una evaluación ciega o cómo se logró^2^, ^14^, ^71^, ^72^ (Recomendación 2).

A partir de nuestra revisión de la literatura, 13 (34%) de los estudios utilizaron un objetivo adicional no entrenado en la terapia para medir la transferencia, lo que demuestra que es un enfoque factible y aceptable para niños, jóvenes y sus familias. Es un enfoque relativamente simple y menos demandante de tiempo para medir la transferencia, y su uso consistente en estudios clínicos y de investigación podría apoyar la agrupación de datos entre estudios (Recomendación 3).

La mayoría de los estudios realizados hasta la fecha han explorado la eficacia (o la eficacia preliminar) del enfoque con diferentes poblaciones cuando se aplica en un entorno de investigación controlado.

Mientras que los estudios de comparación de grupos (RCTs piloto/RCTs) permiten comprender el cambio promedio entre grupos, los estudios de caso único permiten explorar los resultados individuales, como en el caso de varios participantes que logran sus objetivos, ampliando nuestra comprensión de los posibles mejores respondedores.

La efectividad de la intervención CO‐OP cuando es aplicada en la práctica clínica no ha sido explorada en detalle, y sólo unos pocos estudios reportan resultados cuando la intervención es realizada por personal clínico,^17^, ^23^ incluyendo un estudio de ciencia de implementación en cinco unidades de rehabilitación para pacientes hospitalizados con accidente cerebrovascular.^34^, ^73^ Esto es fundamental para comprender si el enfoque puede ser implementado en servicios clínicos con fidelidad y si es aceptable tanto para los profesionales clínicos como para los niños, jóvenes y sus familias (Recomendación 4).

## DIRECCIONES FUTURAS

### Recomendación 1. Diseño de estudios y tamaños de muestra

Se requieren estudios a gran escala, incluidos ensayos controlados aleatorios (RCTs) con el poder estadístico adecuado, para validar la efectividad del enfoque CO‐OP en diferentes poblaciones. Cuando sea apropiado, los estudios de viabilidad con un diseño adecuado deben preceder a ensayos definitivos, de modo que los cálculos de tamaño de muestra se basen en tamaños de muestra lo suficientemente grandes.

Cuando los RCTs no sean prácticos, los estudios de caso único deben incluir suficientes replicas para permitir meta‐análisis, asegurando la consistencia y el rigor de los datos.

### Recomendación 2. Consistencia de la medición para meta‐análisis

La Medida Canadiense del Desempeño Ocupacional y la PQRS son dos medidas recomendadas en el enfoque CO‐OP para evaluar los resultados de la intervención. El uso y la notificación consistentes de estas dos medidas en los estudios facilitarían la futura recopilación de datos para informar sobre los efectos en estudios de meta‐análisis relacionados con la adquisición de habilidades y la transferencia. Se alienta y recomienda la evaluación ciegade la PQRS, ya que es posible implementarlo.

### Recomendación 3. Generalización y transferencia

La evidencia actual respalda la capacidad del enfoque CO‐OP para facilitar la adquisición de habilidades y su aplicación inmediata. Aunque la transferencia no se midió en todos los estudios, se ha demostrado que es uno de los elementos clave que diferencia al enfoque CO‐OP de otras intervenciones. Sugerimos el uso de un cuarto y posiblemente quinto objetivo para medir el efecto de la transferencia de la intervención a objetivos no entrenados.

El uso de análisis económicos de salud para captar los beneficios de la intervención más allá de los objetivos entrenados podría abordar una brecha significativa en el conocimiento para respaldar los servicios clínicos y la financiación de programas.

Además, se necesitan estudios longitudinales para examinar la sostenibilidad de estas habilidades a lo largo del tiempo y su transferibilidad a tareas no entrenadas y nuevos entornos. La evaluación de resultados a largo plazo ayudará a comprender el mantenimiento de las mejoras logradas con la intervención y si se justifican sesiones adicionales en el futuro.

### Recomendación 4a. Estudios de ciencia de implementación y fidelidad

La literatura carece de información sobre la capacitación, la fidelidad y la investigación en implementación. A medida que el enfoque CO‐OP se difunde en entornos clínicos y educativos más amplios, es crucial realizar investigaciones sobre los métodos de capacitación para terapeutas y educadores. Los estudios de ciencia de implementación pueden identificar mejores prácticas para la formación, mecanismos de apoyo para el desarrollo profesional continuo y estrategias para superar las barreras de implementación en diversos contextos. Los estudios de capacitación e implementación son necesarios para garantizar la escalabilidad del enfoque CO‐OP, explorar las mejores prácticas para la capacitación de clínicos y evaluar la fidelidad de la intervención en contextos del mundo real.

### Recomendación 4b. Dosificación de la intervención y formatos de entrega

La dosificación y los formatos óptimos para las intervenciones CO‐OP no han sido explorados de manera sistemática. Aunque el enfoque estándar implica 10 sesiones, se deben explorar variaciones en la frecuencia, la duración y los formatos grupales frente a los individuales para determinar los modelos más efectivos y eficientes. Comprender estas variables puede conducir a planes de intervención más personalizados y accesibles, reduciendo potencialmente la carga para las familias y los sistemas de salud.

### Recomendación 5. Mecanismos subyacentes del cambio

Faltan estudios que identifiquen los ingredientes activos y los mecanismos de cambio, incluidos los resultados relacionados con la transferencia, tal como se definió anteriormente. Para comprender el mecanismo de acción del enfoque CO‐OP, es necesario identificar los procesos activos y/o componentes del tratamiento y probar cómo conducen a resultados positivos. Comprender por qué funciona CO‐OP, a través de qué procesos y cómo ocurre el cambio en diferentes poblaciones, permitiría entender los mecanismos de cambio terapéutico.

Comprender si existen mediadores y moderadores específicos de la eficacia ayudará a desentrañar los mecanismos subyacentes de acción. La evidencia en neurociencias, como la comprensión de las vías de la actividad motora y cómo actúa CO‐OP en términos de vías neuronales e interacciones, podría ser útil para refinar la intervención y proporcionar tratamientos personalizados. Comprender el impacto de CO‐OP en el neurodesarrollo y en las vías cognitivas‐motoras, especialmente en niños con discapacidades complejas, podría perfeccionar el enfoque y orientar hacia tratamientos más personalizados.

## CONCLUSION

Esta revisión narrativa indica que la literatura sobre el enfoque CO‐OP se ha expandido de varias maneras importantes desde su introducción en 2001. En conjunto, la literatura indica que CO‐OP puede apoyar eficazmente la adquisición de habilidades, la generalización y la transferencia en diversas poblaciones, tanto en niños como en adultos, abarcando una amplia gama de objetivos utilizando diferentes estrategias. La literatura podría mejorarse mediante el uso de diseños de investigación más robustos y la incorporación de investigaciones sobre capacitación, fidelidad e implementación.
